# DoMINO: Donor milk for improved neurodevelopmental outcomes

**DOI:** 10.1186/1471-2431-14-123

**Published:** 2014-05-13

**Authors:** Sharon Unger, Sharyn Gibbins, John Zupancic, Deborah L O’Connor

**Affiliations:** 1 Mount Sinai Hospital and the University of Toronto, 600 University Avenue, 19-231, Toronto, Ontario, M5G 1X5, Canada; 2 Trillium Health Partners, 2200 Eglinton Ave West, Mississauga, Ontario, L5M 2 N1, Canada; 3 Beth Israel Deaconess Med Center, Neonatology, Rose 318 330 Brookline Ave, Boston, MA 02215, USA; 4 University of Toronto and The Hospital for Sick Children, 327 Fitzgerald Building, 150 College Street, Toronto, Ontario, M5S 3E2, Canada

**Keywords:** Human milk, Donor milk, Neurodevelopment, Very low birth weight infants

## Abstract

**Background:**

Provision of mother’s own milk is the optimal way to feed infants, including very low birth weight infants (VLBW, <1500 g). Importantly for VLBW infants, who are at elevated risk of neurologic sequelae, mother’s own milk has been shown to enhance neurocognitive development. Unfortunately, the majority of mothers of VLBW infants are unable to provide an adequate supply of milk and thus supplementation with formula or donor milk is necessary. Given the association between mother’s own milk and neurodevelopment, it is important to ascertain whether provision of human donor milk as a supplement may yield superior neurodevelopmental outcomes compared to formula.

Our primary hypothesis is that VLBW infants fed pasteurized donor milk compared to preterm formula as a supplement to mother’s own milk for 90 days or until hospital discharge, whichever comes first, will have an improved cognitive outcome as measured at 18 months corrected age on the Bayley Scales of Infant Development, 3^rd^ ed. Secondary hypotheses are that the use of pasteurized donor milk will: (1) reduce a composite of death and serious morbidity; (2) support growth; and (3) improve language and motor development. Exploratory research questions include: Will use of pasteurized donor milk: (1) influence feeding tolerance and nutrient intake (2) have an acceptable cost effectiveness from a comprehensive societal perspective?

**Methods/Design:**

DoMINO is a multi-centre, intent-to-treat, double blinded, randomized control trial. VLBW infants (n = 363) were randomized within four days of birth to either (1) pasteurized donor milk or (2) preterm formula whenever mother’s own milk was unavailable. Study recruitment began in October 2010 and was completed in December 2012. The 90 day feeding intervention is complete and long-term follow-up is underway.

**Discussion:**

Preterm birth and its complications are a leading cause long-term morbidity among Canadian children. Strategies to mitigate this risk are urgently required. As mother’s own milk has been shown to improve neurodevelopment, it is essential to ascertain whether pasteurized donor milk will confer the same advantage over formula without undue risks and at acceptable costs. Knowledge translation from this trial will be pivotal in setting donor milk policy in Canada and beyond.

**Trial registration:**

ISRCTN35317141; Registered 10 August 2010.

## Background

Technological advances in the neonatal intensive care unit (NICU) have greatly enhanced the survival rate of very low birth weight (VLBW) infants (<1500 g). In developed countries, more than 90% of infants born <32 weeks gestation now survive initial hospitalization [1]. Coincident with this improved survival, VLBW birth is an important cause of long-term neurological morbidity in childhood. Interventions which reduce morbidity and promote normal brain development for VLBW infants are thus urgently required [2,3].

### Neurodevelopmental outcomes of VLBW infants and the role of mother’s own milk

Many VLBW infants show continued neurologic sequelae such as cognitive deficits, academic underachievement, grade failures and the need for remedial assistance during childhood and middle adolescence [3]. Aside from intracranial lesions associated with preterm birth, factors shown to impact the neurodevelopmental outcome of VLBW infants include gestational age at birth, sepsis, necrotizing enterocolitis (NEC), chronic lung disease, suboptimal nutrient intake or poor growth [2-8]. Compelling evidence exists to suggest that use of mother’s own breastmilk compared to infant formula during initial hospitalization positively affects the neurodevelopment of VLBW infants during early childhood and beyond [9-15]. Vohr et al. demonstrated a dose dependent relationship between breastmilk intake for extremely low birth weight infants enrolled in the National Institute of Child Health and Human Development Glutamine Trial. For every 10 mL/kg/day increase in breastmilk intake, there was an associated increase in the Bayley Mental Development Index of 0.53 at 18 months corrected age (CA) [14] which persisted to 30 months CA [15]. The impact of this can be seen to be quite dramatic when comparing a baby who received no breastmilk to one who received 150 mL/kg/day which would equate to a difference of 7.5 points.

Mother’s own milk is thought to improve neurodevelopment because it is well tolerated by the VLBW infant, facilitating the transition from parenteral to enteral feeding, and due to its nutrient composition (with fortification as appropriate); both factors resulting in the provision of optimal substrate for brain development. Additionally, mother’s milk, via a myriad of bioactive components such as secretory IgA, lactoferrin and growth factors, protects from morbidities associated with preterm birth (NEC, sepsis, other infections) that are in turn additive risk factors for an adverse neurological outcome [9,15,16].

### Use of mother’s own milk in the VLBW population

The World Health Organization, the American Academy of Pediatrics and the Canadian Paediatric Society all recommend mother’s own milk as the exclusive source of feeding for infants during their first 6 months of life [17-19]. Despite the many advantages of feeding mother’s own milk, the majority of mothers of VLBW infants, for a variety of reasons such as illness, stress, mammary secretory cell immaturity and other factors related to preterm birth, are unable to express adequate amounts of milk to exclusively feed their children [20]. A smaller percentage of mothers choose not to pump their breasts to provide breastmilk.

### Use of pasteurized donor human milk

In North America, preterm formula is increasingly being replaced by pasteurized donor human milk when a supplement to mother’s own milk is required for VLBW infants. Currently there are three non-profit donor milk banks in Canada and eleven in the United States with more in the planning stages [21]. There is currently one for profit company in the United States that partners with NICUs to provide donor milk. There remains however a paucity of scientific research with respect to pasteurized donor milk use in the NICU.

In a Cochrane systematic review and meta-analysis, Quigley et al. demonstrated both benefits and risks associated with the use of donor milk [22]. See Table 1 for a summary of the individual studies included in this review. Importantly, there was a higher incidence of NEC among infants with birth weights < 2500 g and fed formula versus those fed donor milk (relative risk of 2.5 [95% CI, 1.2, 5.1]). Because NEC is the most common gastrointestinal emergency among VLBW infants, its prevention is a powerful argument in favor of donor milk as an alternative supplement to formula when mother’s own milk is not available [23]. NEC may lead to perforation of the bowel, bowel resection and death or long-term feeding problems associated with a shortened gastrointestinal tract [23]. Further, NEC, particularly surgical NEC has been shown to be associated with adverse neurodevelopment outcomes at 18–24 months CA [24].

**Table 1:** Formula milk versus donor milk for feeding preterm or low birth weight infants (Cochrane review)

**Author**	**Year**	**Subjects**	**Comparison**	**Blind**	**Primary outcome**	**Notes**
**Davies**[25]	1977	68 preterm (28–36 weeks)	Term formula vs Donor Milk	No	Slower growth first month for Donor Milk	Uncertain group for 2 infants with mother’s own milk
**Gross**[26]	1983	67 preterm (27–33 weeks)	Term formula vs Donor Milk	No	Slower growth for term Donor Milk (not preterm Donor Milk)	Infants with feed intolerance/NEC withdrawn from growth analysis
**Lucas**[27]	1984	159 LBW (<1850 g)	Preterm formula vs Donor Milk	No	Slower growth for Donor Milk; no neurodevelopmental difference	
**Lucas**[28]	1984	343 LBW (<1850 g)	Preterm formula vs Donor Milk	No	No neurodevelopmental difference	
**Raiha**[29]	1976	106 LBW (<2100 g)	Term formula vs Donor Milk	No	No difference in growth	
**Schanler**[30]	2005	173 preterm (<30 weeks)	Term formula vs Fortified Donor Milk	Yes	Slower growth for Donor Milk, no difference in infection events	Only fortified Donor Milk study; 20% cross-over from Donor Milk to Formula
**Schultz**[31]	1980	20 preterm	Term formula vs Donor Milk	No	No difference in weight gain	
**Tyson**[32]	1983	81 LBW (<1500 g)	Preterm formula vs Donor Milk	No	Slower growth for Donor Milk	Donor Milk not pasteurized; Randomized day 10; 5 affected infants withdrawn

Quigley MA, Henderson G, Anthony MY, McGuire W. Formula milk versus donor breast milk for feeding preterm or low birth weight infants. Cochrane Database Syst Rev 2007:CD002971.

The Quigley et al. review and meta-analysis, however, concluded that infants fed donor milk experienced slower weight (*p* < 0.0001), length (*p* < 0.0003) and head circumference (*p* < 0.0001) gains than those fed formula. These risks associated with donor milk are of significant concern because VLBW infants are born with impoverished nutrient reserves, and are subject to metabolic stresses that further elevate nutritional requirements [22,23]. Nutrient deficits and sub-optimal growth has significant long term neurodevelopmental consequences [2,23,24,33,34].

Quigley et al. point out that all but one of the randomized controlled trial (RCTs) examined in their meta-analysis were >25 years old when smaller VLBW infants did not survive. Feeding practices have also since changed to include preferential use of mother’s own milk along with nutrient fortification of human milk to promote adequate growth. There was only one RCT [30] in the Cochrane review that was reflective of current clinical practice which includes much smaller babies, preferential use of mother’s own milk and nutrient fortification. This study however did not look at long-term neurocognitive outcomes.

### Effects of the pasteurization process

The donor milk used in North American NICUs is typically pooled from 3 or more mothers to reduce batch-to-batch variability in nutrient composition and is pasteurized (Holder pasteurization, 62.5°C for 30 minutes) according to Human Milk Banking Association of North America (HMBANA) guidelines to prevent the transmission of infectious agents (e.g. HIV, pathogenic bacteria [35]). Despite pooling, donated milk often contains a lower concentration of energy, protein, fatty acids and other nutrients compared to mother’s own milk due to the fact that the donations usually come from mothers who deliver a healthy term infant several weeks or months after delivery once they have accrued a surplus of pumped milk [36-38]. It is well known that the concentration of a number of nutrients, most especially protein, are higher in the breastmilk of mothers delivering preterm compared to that of mothers of term born infants [39,40]. Further, the concentration of many nutrients in breastmilk decline with the progression of lactation [39]. While many nutrients are unaffected, Holder pasteurization will impact the concentration of some nutrients in breastmilk, most notably a number of the water soluble vitamins (e.g. folate and vitamin C) [41,42] (Table 2). Further, donor milk, compared to mother’s own milk, also undergoes at least one additional collection/storage container transfer and freeze-thaw cycle, as part of the pasteurization process affecting the concentration of many nutrients in breastmilk as fat adheres to the walls of each vessel [39].

**Table 2:** Breastmilk components and the effect of pasteurization

**Component**	**Effect**	**References**
Adiponectin	33% reduction	[43]
Amylase	15% loss of activity	[44]
B-cells, T-cells	Abolished	[45,46]
Bile salt dependent lipase	Abolished	[44]
CD14 (soluble)	88% reduction	[47]
Fats:		
Total fat	No effect	[48-50]
C14:1-C24:1	No effect	[44,49]
C8:0-C24:0	No effect	[44,49]
n-3,n-6 PUFA	No effect	[44,49]
AA, DHA	No effect	[44,49]
Linoleic, linolenic	Reduced	[51]
Calcium	No effect	[48]
Copper	9% reduction No effect	[50,52]
Escherichia coli inhibition	26% reduction	[53]
Epidermal growth factor	No effect	[54,55]
Erythropoeitin	Significantly reduced	[56]
Folate	16-31% reduction	[41,42]
Free fatty acids	80% increase	[57]
Gangliosides	No effect	[54]
Hepatocyte growth factor	60% reduction	[54]
Immunoglobulins:		
IgA, sIgA	0-48% reduction	[45,46,48,58-62]
IgG	34% reduction	[60]
IgM	Abolished	[61,62]
Insulin	46% reduction	[43]
IGF-1, IGF-2, IGF-BP2,3	7-39% reduction	[55]
IL-1β, IL-10	Significantly reduced	[54,56]
IL-2, Il-4, IL-5, IL-12, IL-13	No effect	[54]
IL-8	Increased	[54]
Interferon gamma	Significantly reduced	[54]
Iron	0-15% reduction	[50,52]
Lactate	7% reduction	[57]
Lactoferrin	57-80% reduction	[58,60,61]
Lactose	No effect	[48,50]
Lipoprotein lipase	Abolished	[44]
Lysine	Significantly reduced	[61,63]
Lysozyme activity	No effect 24-60% reduction	[58-62]
Lymphocytes	Abolished	[46]
Magnesium	No effect	[48]
Mannose-binding lectin	No effect	[47]
Oligosaccharides	No effect	[64]
Phosphorus	No effect	[48]
Potassium	No effect	[48]
Protein	No effect Reduced	[43,48,50,62]
Sodium	No effect	[48,50]
TGF-α, TGF-β	No effect Reduced	[54,65]
Vitamin A	No effect	[50]
Vitamin B6	15% reduction	[42]
Vitamin C	36% reduction	[42]
Zinc	0-3% reduction	[50,52]

Modified with permission from: Ewaschuk JB, Unger S, Harvey S, O’Connor DL, Field CJ. Effect of pasteurization on immune components of milk: implications for feeding preterm infants. Applied Physiology, Nutrition, and Metabolism 36:175–182, 2011.

Very little is known about the impact of the pasteurization process on the myriad of bioactive components in human milk, many of which serve a dual role in nutrient absorption and as anti-infective agents [66]. Holder pasteurization reduces the concentration of immunoglobins (secretory IgA, G and M) [48,58,67]. Live cellular components, including B and T lymphocytes are eliminated (Table 2). Of the few enzymes that have been studied, milk amylase is relatively unaffected, lysozyme in more recent reports has been shown to be affected and lipases are completely denatured [44,48,58,59,67]. Fat absorption is lower in donor versus mother’s own milk-fed infants, presumably due in part to destruction of bile salt stimulated lipase known to compensate for low intraluminal lipase activities necessary for fat absorption [68]. Lactoferrin, an anti-microbial and immunomodulatory iron-binding glycoprotein shown in both animal studies and a recent clinical trial to be effective against neonatal sepsis is reduced by 80% [58,69,70]. Immune modulators known to be important in NEC prevention such as IFN-γ, TNF-α, IL-1β. IL-10 and hepatocyte growth factor remain present in donor milk post pasteurization however in significantly reduced quantities [54].

### Study objectives

The primary question is whether the use of pasteurized donor milk, compared to preterm formula, as a supplement to mother’s own milk for the first 90 days after randomization or until hospital discharge, whichever comes first, improves cognitive development of VLBW infants at 18 months corrected age as measured by the Bayley Scales of Infant Development, 3^rd^ edition (BSID). Secondary questions are whether donor milk will reduce a composite of death and serious morbidity (NEC, late onset sepsis, chronic lung disease and severe retinopathy of prematurity); support growth; and improve language and motor development at 18 months corrected age. Exploratory research questions include: will use of donor milk, as a supplement to mother’s own milk: (1) influence feeding tolerance and nutrient intake (2) have an acceptable cost effectiveness (medical and non medical) from comprehensive societal perspective?

## Methods/Design

This is a pragmatic multi-centre, double-blind, RCT designed to evaluate the effectiveness of pasteurized donor milk as a supplement to mother’s own milk in those infants when mother’s own milk is unavailable. The analysis will be conducted using an “intention to treat” approach. Infants randomized to the intervention group received donor milk when mother’s own milk was unavailable. Infants randomized to the control group received formula designed for preterm infants when mother’s own milk was unavailable.

Funding was received from the Canadian Institutes for Health Research (MOP#210093), SickKids Foundation and the Ministry of Health and Longterm Care of Ontario. Infants were recruited in one of four participating level III NICU in Toronto and Hamilton, Canada. A comprehensive list of parental and infant demographic variables was collected after written informed consent was secured (Table 3). Randomization, performed within 96 hours of birth, was done centrally using a 24 hr/day web-based third party randomization service (Centre for Mother Infant and Child Research, Toronto). The study allocation was randomly assigned in a ratio of 1:1, in random blocks of 4 and 8, with stratification by centre and birth weight grouping (<1000 g and 1000–1499 g). Aside from the research dietary technicians assigned to the study and a single neonatal dietitian, all members of the research study team (including outcome assessors), clinical teams and families are blinded to study allocation.

**Table 3:** Demographic variables collected during the DoMINO trial

**Infant characteristics**	**Prenatal and parental characteristics**
Gestational age at birth+	Gravity/Parity*
Birth weight, length and head circumference+	Artificial reproductive technology (type and origin of eggs, sperm)+
Size for gestational age (small [SGA], appropriate [AGA] or large for [LGA] gestational age)+	Antibiotic use prior to delivery (prior 2 weeks)+
Sex+	Use of Prenatal Steroids+
Multiple birth status+	Cesarean delivery*
5-minute Apgar score+	Mom has previously breastfed (yes/no)*
Newborn Illness severity score (SNAP-II+)	Mom intends to breastfeed (yes/no)*
	Parental education (highest degree or diploma attained)*
	Parental weight and height (self-reported)*
	Parental age*
	Number of children in current household*
	Smoking (maternal history during pregnancy)*
	First language spoken in the home*
	Socioeconomic status (single parenting; above or below poverty line)*
	Ethnicity*

Baseline demographic variables collected by means of parental interviews* or from medical records +.

The feeding intervention continued for 90 days including transfer to a participating level II unit or until discharge home, whichever occurred first. Other than whether infants received donor milk or preterm formula as a supplement, all other aspects of feeding were directed by the clinical team at each NICU. General feeding guidelines were agreed upon by the participating NICUs to provide a consistent approach, and to set criteria necessitating removal from the feeding protocol. These detailed guidelines are found in Table 4. Donor milk was purchased and shipped primarily from the Mother’s Milk Bank of Ohio (>95%) with backup from Calgary Mother’s Milk Bank, both HMBANA members. Donor milk from Ohio was collected and pooled from at least three women who had delivered within the previous three months. Once nutrient fortification of donor milk commenced, a protein module (0.3 g/dl, Beneprotein, Nestle) was added to donor milk to bring the analyzed protein content (0.9 g/dl) up to the average estimated protein concentration of mother’s own milk after 30 days (1.2 g/dl) [71,72].

**Table 4:** Feeding guidelines

	**Supplement to MOM**	**Supplement to MOM**
	Pasteurized Human Donor Milk	Preterm Formula*
		Similac Special Care 20 or 24 kcal/ oz [3.0 g protein/100 kcal] [Abbott Laboratories] or
		Enfamil Premature Formula 20 or 24 kcal/oz [3.0 g protein/100 kcal] [Mead Johnson Nutritionals]
**Initiation of enteral feeding**	Day 1-7	Day 1-7
**Volume of feeding at initiation**	10-20 ml/kg/d (hold volume for 3-5 days)	10-20 ml/kg/d (hold volume for 3–5 days)
**Rate of feed advancement**	10-25 ml/kg/day	10-25 ml/kg/day
**Fortification to commence at >120 ml/kg/day using milk pumped > 7 days after parturition+**	Human milk fortifier	Not Applicable
	Enfamil Human Milk Fortifier, [Mead Johnson Nutritionals] or	
	Similac Human Milk Fortifier, [Abbott Laboratories]	
**Volume**	140-200 ml/kg/d to achieve a weight gain of > 15 g/kg/day	140-200 ml/kg/d to achieve a weight gain of > 15 g/kg/day
**Minimum protein dose to be provided once (or up to 3 days after) volume reaches >150 ml/kg/d**	3.0 g/kg/d**	3.0 g/kg/d**
**At 24 kcal/oz and weight gain < 15 g/kg/d for 3–7 days**	Concentrate feeding using a multi-nutrient modular to 26–27 kcal/oz	Modular to 26–27 kcal/oz
**At 27 kcal/oz and weight gain < 15 g/kg/d for 3–7 days**	Concentrate feeding using a multi-nutrient modular to 30 kcal/oz	Concentrate feeding using a multinutrient modular to 30 kcal/oz
**At 30 kcal/oz and weight gain < 15 g/kg/d for 3–7 days**	Remove from feeding protocol	Remove from feeding protocol

*A low or high iron formula will be used for study infants at each NICU depending on the iron content of the human milk fortifier in use to avoid unblinding of feeding assignments.

+Commence supplementation with 200–400 IU vitamin D and 2–4 mg/kg/d elemental iron.

**As infants approach their estimated date of delivery and/or readied for hospital discharge intakes will usually be below this value.

Each morning, a member of the health care team at each NICU completed and FAXed in a feeding order for study infants in their care to our research diet technicians. All study feeds for research participants were prepared in a single milk preparation room at the Hospital for Sick Children in a laminar flow cabinet. Feeds were delivered to units daily in amber single-use oral syringes (Baxa, Deerfield Illinois) or orange plastic wrapped bottles by study staff or courier. The contents of syringes and bottles were indistinguishable by visual inspection.

**Figure 1:**
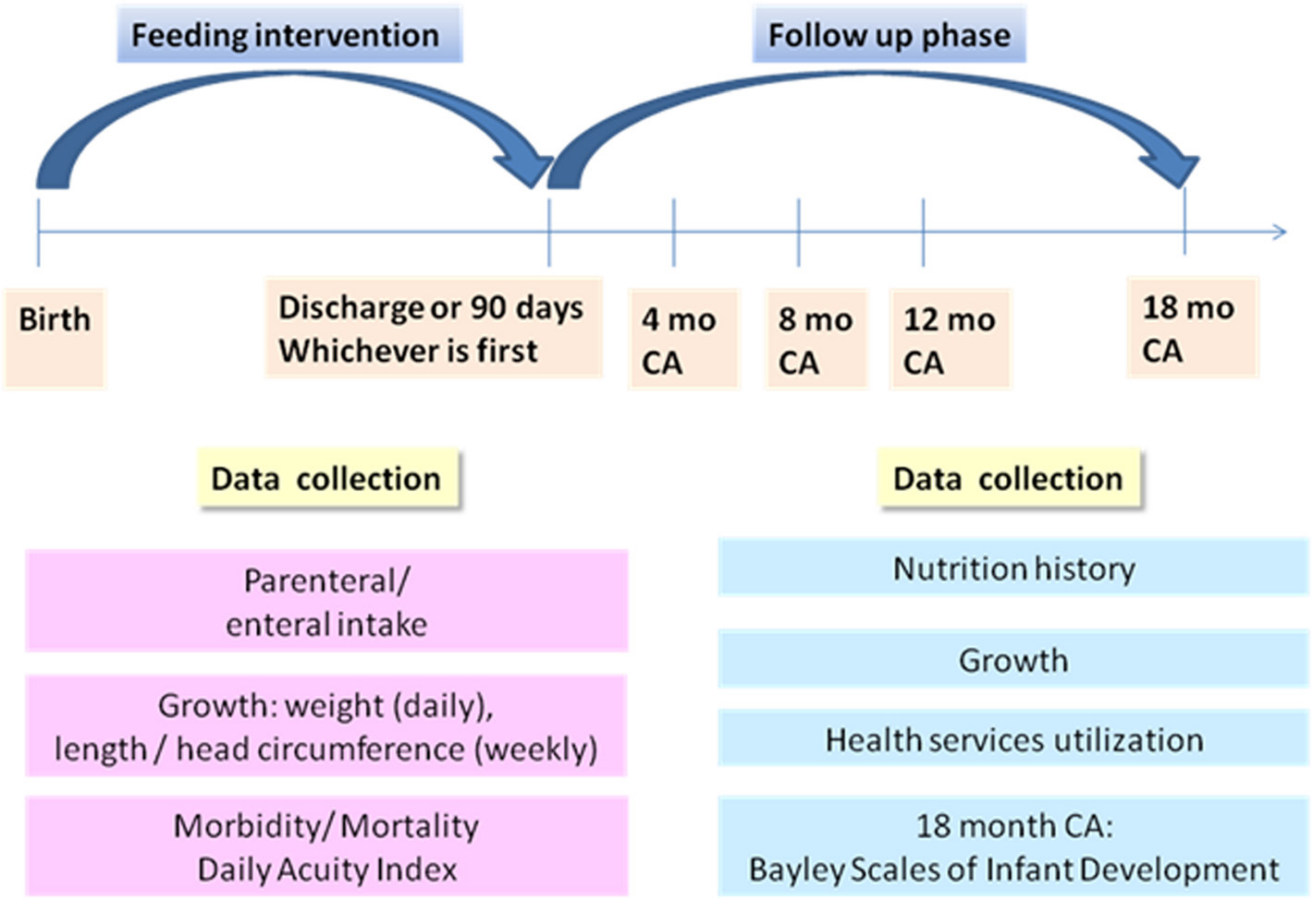
Frequency and duration of follow up.

The frequency and duration of assessment during the feeding intervention and after hospital discharge are illustrated in Figure 1. During the feeding intervention, morbidity/mortality, growth, feeding tolerance, nutrient intake data and level of respiratory support (daily acuity index proxy) were extracted prospectively from the medical record or directly assessed on a weekly basis by a member of the study team. Following discharge home, infants are seen at clinic visits scheduled at 4, 8, 12 and, 18 months CA corresponding to important feeding, growth and developmental milestones. Additionally, families are called monthly after discharge to ascertain healthcare resources accessed on behalf of the child (e.g. visiting a pediatrician, home care). During these calls, information on current feeding practices is collected as these (e.g. duration of breastfeeding) may influence neurodevelopment.

A review of safety data (growth, major morbidity) occurred after one- and two-thirds of infants completed the feeding intervention by an external Data Safety and Monitoring committee. The study was approved by the Research Ethics Committee at each participating hospital.

### Inclusion/exclusion criteria

**Figure 2:**
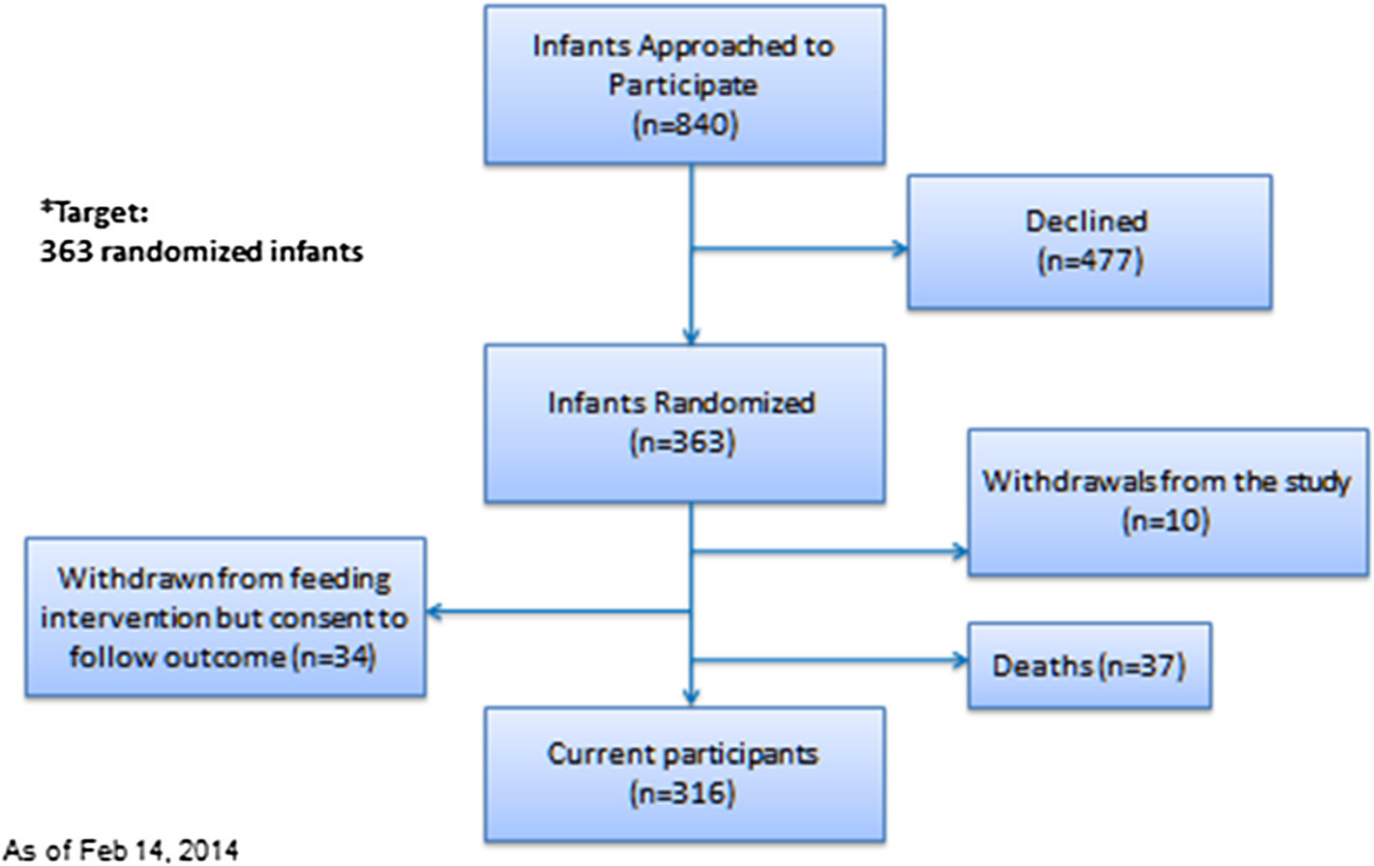
Subject disposition to date.

The inclusion criteria were (1) day 1 to 4 of life; (2) <1500 g birth weight; (3) enteral feeding expected to be initiated in the first 7 days of life. The exclusion criteria were (1) infants with serious congenital or chromosomal anomalies that may contribute to poor developmental outcome; (2) severe asphyxia; (3) enrolment in any other clinical study affecting nutritional management during the feeding intervention; (4) reasonable potential that the infant would be transferred to a NICU where the study protocol could not be continued. The study feeding protocol was stopped if (1) the infant died during the intervention period; (2) a parent(s) requested withdrawal from the feeding protocol; (3) the infant had inadequate weight gain despite nutrient concentration of feedings (4) there was a requirement for thickening of feeds that would unmask the feeding assignment. In the event that an infant was withdrawn from the study feeding protocol, the family was requested to allow Research Staff to continue data collection and to complete the follow-up phase after discharge. See Figure 2 for a review of subject disposition to date.

### Outcome measures

The primary outcome for this trial is the cognitive composite score on the BSID-III at 18 months CA. The BSID-III is a standardized test designed to assess the cognitive, language and motor (fine, gross) development of infants from 1 to 42 months of age [73]. A decision was made to administer this test at each infant’s 18 month CA birth date because of the improved predictive validity at this time compared to earlier ages [73,74].

The secondary mortality/morbidity composite comprised death and major morbidity including NEC, late onset sepsis, chronic lung disease or severe retinopathy of prematurity. Late onset sepsis was defined as a positive culture from blood, cerebrospinal fluid, catheter or suprapubic urine at >5 days after birth. A confirmed case of NEC was defined as stage 2 or 3 by the Modified Bell Staging Criteria [75]. Infants were classified as having chronic lung disease as assessed at 36 weeks postconception by NIH criteria [76]. Severe ROP (Stage 4 or 5) was defined according to International criteria or if laser surgery or intraocular anti-vascular injection was required [77-79].

Weight (+/-2 g), length (+/-0.1 cm) and head circumference (+0.1 cm) were measured weekly during hospitalization and at routine post-discharge clinic visits using standardized procedures and precision equipment as previously described [80,81]. To account for the different GA of infants, z-scores for anthropometric measurements are computed using the Fenton preterm growth charts and the World Health Organization Growth Standard after 50 weeks gestational age [82]. Changes in weight as a consequence of the intervention will be specifically examined in relation to gains in length and head circumference as there is little evidence that weight gain alone (i.e. fat mass gain) will benefit the long-term outcome of infants.

Exploratory variables include feeding tolerance and nutrient intakes during the feeding intervention. The volume and estimated energy/nutrient density of study milk and other sources of nutrition (e.g. parenteral nutrition, mother’s own milk, vitamin supplements) were extracted prospectively from medical records by study staff during the feeding intervention. Feeding tolerance will be assessed by describing the days to full enteral feeding (150 ml/kg/d) and number of days feedings were withheld.

In addition, a health economic analysis of the use of pasteurized donor milk compared to preterm formula as a supplement to mother’s own milk is being conducted. The specific objectives of the health economic analyses are:

(i) To measure and compare the relevant health and non-health costs of neonatal care to 18 months, for VLBW infants fed donor milk or preterm formula as a supplement.

(ii) As appropriate, use measured costs in conjunction with the efficacy data from the clinical trial to estimate the cost per five-point improvement in BSID-III through use of donor milk in VLBW infants.

(iii) To use decision-analytic modeling and secondary literature sources to estimate the long-term health and non-health costs, as well as long-term quality of life outcomes and cost per quality adjusted life year, for enrolled infants, based on outcomes measured to 18 months CA.

All relevant health economic data is collected prospectively monthly from the time of study enrolment until 18 months CA.

Although some differences in resource utilization between treatment and control groups will be related to the costs of donor milk and potentially to differential neonatal therapeutic requirements if one group has improved growth or fewer adverse events such as NEC, there may be longer-term differences in costs if donor administration is associated with improvements in neurodevelopment [83]. Unfortunately, the measurement of cost-related outcomes beyond the 18 month endpoint for the clinical trial data collection is not feasible. Instead, a decision-analytic model will be constructed to synthesize longer-term cost effectiveness estimates in a secondary analysis [84]. In order to balance the importance of long-term cost-effectiveness with the potentially reduced validity of literature-supplemented cost estimates, results will be reported primarily in terms of measured cost-effectiveness to 18 months, and secondarily in terms of modeled cost-effectiveness through the lifetime. The former results will be of interest primarily to hospital decision makers and third-party payers, while the latter will more strictly maintain the societal perspective important to the broader clinical and policy-making audience.

### Statistical analyses

Using a 5 point difference in composite cognitive scores at 18 months CA, with 80% power (alpha level of 0.05), and an estimated standard deviation in each feeding group of 15, we estimated that we required 142 infants in each feeding arm. We assumed a 10% loss to follow-up during the feeding intervention and an additional 10% loss to follow-up after discharge, thus necessitating 176 infants to be randomized in each of the two feeding groups. As the number of infants withdrawn from the study exceeded 10%, primarily due to infant death, we overenrolled by 11 subjects to produce a final sample size of 363 subjects.

Analyses will be carried out using SAS Version 9.1 (SAS Institute, Cary, NC, USA). Descriptive statistics will be calculated for all variables of interest. Continuous measures will be summarized using means and standard deviations whereas categorical measures will be summarized using counts and percentages. The primary analysis for this intent-to-treat study will include all infants as randomized, regardless of adherence with the feeding protocol. The initial analysis will be a comparison of means (cognitive composite score) between the 2 groups at 18 months CA. A generalized linear model will carry out this comparison, controlling for length of treatment and adjusting for correlation of observations taken at the same centre. Sex, birth weight strata (<1000, 1000–1499 g), use of antenatal steroids, multiple birth, a count of key morbidities and duration of mother’s own milk-feeding are prognostic of neurodevelopmental outcome, and for this reason we will perform a secondary analysis which will include a regression model adjusted for the aforementioned characteristics [4,6,13].

Children who cannot be tested on the BSID-III due to disability, severe delay or who perform below the threshold of the test for individual composite scores (cognitive, language motor) will be assigned a score of 49 [7]. The scores will be computed by trained individuals who have established inter rater reliability (R^2^ = 80) and who are blind to study group. Other missing data will be handled using methods of multiple imputation.

## Discussion

More than 2,700 VLBW(<1500 grams) babies are born in Canada each year with a length of hospital stay varying from 59 to 113 days compared to an average of 2–3 days for a healthy term infant [85,86]. Although children born of VLBW represent a small proportion of children born in Canada, they represent an important cause of neurodevelopmental delay and disability in childhood and consume a disproportionately large amount of health care dollars [3,85,87]. Interventions that can promote better health and development of these children can thus have a large impact. Although, in older trials, donor milk was protective against the development of NEC, there have been no studies to date assessing long-term outcome following donor milk supplementation in current era NICUs.

The societal implications of a 5 point difference in the cognitive composite score on the Bayley Scales of Infant and Toddler Development (BSID) are substantial [15]. Currently, half of VLBW infants require special education services at school [88-91], even among children without neurosensory impairment [92] which has been shown to translate into a lower level of academic achievement in adulthood (e.g. number of high school graduates) [93,94]. Vohr et al. argue that a 5 point difference could translate into a reduction in the number of children requiring special education services, associated costs, and improve long-term academic achievement [15]. In a detailed analysis of the economic gains realized as a result of lowering environmental lead exposure in the U.S., Grosse et al. estimated that the 5 point improvement in cognitive scores realized amongst 1–5 year old children equates to a 9 to 12% increase in work productivity in adulthood [83].

Anderson et al. previously showed a 5 point improvement in cognitive outcome in low birth weight infants fed mother’s own milk instead of formula [9]. Given the VLBW infants in this RCT were considerably smaller than those in the Anderson meta-analysis, it is reasonable to assume our effect size may be larger as serious morbidity (e.g. NEC and sepsis), poor growth, and long-term neurodevelopmental sequelae are inversely related to gestation at birth [2,3]; hence, there is a much greater opportunity for human milk to improve BSID-III scores.

The 90 day intervention period was chosen as at the time of initiation of this trial, the only available RCT on this research question reflective of current clinical practice also used a 90 day period [30]. This will facilitate a comparison of results from the two studies. Ninety days also reflected the average length of hospital stay of VLBW infants from the recruiting NICUs at the study initiation. The duration of the exposure is sufficient to observe differences in neurodevelopmental outcome at 18 months as Lucas et al. demonstrated differences in BSID scores at 18 months CA between infants (<1850 g) fed mother’s own milk versus formula for an average duration of 4 weeks during initial hospitalization [74]. These differences translated into a higher IQ (8.3 points) at 7.5 years of age even after controlling for maternal education and social class (*p* < 0.0001).

### Health economic impact

It has been estimated that the incremental direct medical costs of preterm birth in the United States exceed 26 billion dollars [93,94]. Despite these significant tallies, most economic analyses in the literature have focused on the global package of neonatal care or on new technologies or therapies such as surfactant, nitric oxide or erythropoietin. However, less glamorous components in the treatment of VLBW infants, such as optimal feeding, may also involve significant expenditures due to their frequency of use. It is important in an era of increasing financial constraint to establish not only the evidence for clinical efficacy of such therapies, but also the evidence for their value-for-money. Furthermore, if such economic information is to conform to the standards of evidence used for clinical efficacy and remain free of systematic bias, it is essential that data are collected prospectively, in conjunction with RCTs, whenever possible. Although one study has used modeling techniques to estimate the cost implications of the use of donor milk [95,96] and a second report examined the costs of preventing necrotizing enterocolitis in an exclusive human milk diet including human based fortifier [97], there has been no direct measurement of cost of donor milk use alongside a randomized trial or other strong study design. The establishment of a donor milk bank requires a substantial commitment of resources, due to the volume of product handled and the need for rigorous safeguards against contamination, misidentification or infection. The costs of avoiding these risks may, indeed, be of a similar magnitude to blood banking. Conversely, if the use of donor milk lowers the incidence of adverse consequences such as NEC or improves growth or neurodevelopmental status, the costs for the intervention group may be lower in the medium or long-term. The balance of short-term and long-term costs and savings can only be estimated through formal economic evaluation.

Results of this pragmatic trial will determine the most effective supplement for VLBW infants when mother’s own milk is unavailable to support neurocognitive development. We anticipate data from this study, including a health economic analysis, will immediately impact the development of milk banks in Canada and beyond.

## Abbreviations

BSID-III: Bayley Scales of Infant Development, 3^rd^ edition; CA: Corrected age; DoMINO: Donor milk for improved neurodevelopmental outcomes; HMBANA: Human milk banking association of North America MOM, Mother`s own milk; NIH: National Institutes of Health; NEC: Necrotizing enterocolitis; NICU: Neonatal intensive care unit; PDM: Pasteurized donor milk; ROP: Retinopathy of Prematurity; VLBW: Very low birth weight.

## Competing interests

SU is the medical director of a human milk bank for which both SU and DLO are the co-chairs of the Advisory Board for the milk bank. All authors declare no financial competing interests.

## Authors’ contributions

SU, SG and DLO are the primary investigators for the DoMINO trial and thus contributed substantially to the development of the protocol as well as drafting and critically revising the manuscript. JZ is a co-investigator for the DoMINO trial, responsible the health economics evaluation and for critically revising this manuscript. All authors read and approved the final manuscript.

## Pre-publication history

The pre-publication history for this paper can be accessed here:

http://www.biomedcentral.com/1471-2431/14/123/prepub
